# Generation of Mice with Hepatocyte-Specific Conditional Deletion of *Notum*

**DOI:** 10.1371/journal.pone.0150997

**Published:** 2016-03-14

**Authors:** Frédéric Canal, Sara Charawi, Gisèle Grimber, Christophe Houbron, Valérie Drouet, Sabine Colnot, Benoit Terris, Catherine Cavard, Christine Perret

**Affiliations:** 1 Inserm, U1016, Institut Cochin, 75014 Paris, France; 2 Cnrs, UMR8104, 75014 Paris, France; 3 Université Paris Descartes, 75014 Paris, France; 4 Equipe labellisée LNCC, Paris, France; 5 Pathology Unit, Hôpital Cochin, 75014 Paris, France; University of Kentucky, UNITED STATES

## Abstract

**Background:**

Fine tuning of the Wnt/β-catenin signaling pathway is essential for the proper development and function of the liver. Aberrant activation of this pathway is observed in 20%-40% of hepatocellular carcinomas (HCC). *Notum* encodes a secreted Wnt deacylase that inhibits Wnt activity and thereby restricts the zone of activation of Wnt/β-catenin signaling. An important role of *NOTUM* has been described in development in drosophila, planaria and zebrafish, but its role in the mammalian liver is unknown. *Notum* is required for spatial control of the Wnt/β-catenin signaling in several animal models and the Wnt/β-catenin pathway contributes to liver patterning involved in metabolic zonation. Therefore, *Notum* may be involved in the liver patterning induced by the Wnt/β-catenin signaling during the adult stage.

**Methodology/principal findings:**

We generated a conditional *Notum* knockout mouse mutant to study the effect of the deletion of *Notum* in the liver. We show that *Notum* is a direct target of the Wnt/β-catenin signaling in the liver. Liver-specific deletion of *Notum* did not modify liver zonation, but *Notum* deletion had a long-term effect on mouse physiology. In particular, male mutant mice developed metabolic disorders.

**Conclusion:**

We show that *Notum* is not a key actor of Wnt/β-catenin-dependent liver patterning of adult mice, but has role in liver glucose homeostasis. Male mice deficient in *Notum* specifically in the liver develop metabolic dysfunctions implicating *Notum* in the development of Type 2 diabetes.

## Introduction

The morphogenic Wnt/β-catenin signaling pathway is essential in numerous physiological processes during embryonic development and in the maintenance of self-renewing tissues in adulthood [1]. It is a key element of liver biology during hepatic development and regulates metabolic liver zonation in adults [2]. Liver zonation is the specialization of hepatocytes in particular metabolic functions depending on their position within the liver lobule [3]. The demonstration that Wnt/β-catenin signaling governs metabolic zonation by controlling liver patterning in the adult stage was an important breakthrough [4]. β-catenin signaling controls perivenous and periportal programs of hepatocytes in an opposite fashion. Using genetically modified mice with targeted activation or inactivation of β-catenin signaling we identified the genetic program controlled by β-catenin that regulates liver zonation; we proposed a model of how β-catenin transcriptionally controls the complex regulatory network that shapes zonated gene expression in the liver [5]. However, the precise molecular mechanisms by which β-catenin may pattern the liver are still unknown.

Although Wnt/β-catenin signaling is essential for development and adult tissue homeostasis, its constitutive activation is oncogenic in numerous tissues [1]. Activating mutations of *CTNNB1*, the gene encoding β-catenin, are present in 20–40% of hepatocellular carcinomas (HCC), the most frequent primary liver cancer [6], and are the most common oncogenic driver in HCC [7]. In addition, HCC with *CTNNB1* mutations define a particular HCC subtype with well-differentiated histology and better survival than other subtypes [7,8].

*Notum* was first identified in drosophila as a negative regulator of the Wnt/β-catenin pathway, involved in tissue patterning during embryonic development. It is required for the normal development of the wing imaginal disc [9, 10]. In planarian model, *Notum* is required for the polarization of the antero-posterior axis during head regeneration and in zebrafish for the correct development of the neural tube and primary motor innervation [11–13]. In all these models, *Notum* acts through the restriction of Wnt/β-catenin signaling activation in a particular zone, which is necessary for appropriate development or during regeneration after injury. *Notum* encodes a hydrolase and it was suggested initially that it cleaves the GPI anchors in two proteins (Dally and Dally-like protein) that modulate the extracellular distribution of Wnt activity [9]. However, very recent study shows that *Notum* is a Wnt deacylase that acts extracellularly, and removes the palmitoleoylation group from Wnt3a protein thereby rendering the Wnt protein inactive [14, 15].

*Notum* is required for the spatial control of Wnt/β-catenin signaling in several animal models and Wnt/β-catenin is central to adult liver physiology especially for metabolic zonation; consequently, *Notum* may be involved in the mechanism by which Wnt/β-catenin signaling patterns the liver in the adult stage. To test this possibility, we generated a conditional *Notum* knockout mutant in mice to study the effect of *Notum* deletion specifically in the liver. We found that liver-specific deletion of *Notum* did not modify liver zonation, but had a long-term effect on mouse physiology; in particular, male mice with liver-specific deletion of *Notum* developed metabolic disorders with age.

## Materials and Methods

### Ethics Statement

#### Human samples

The serie of surgically resected primary liver tumours (Cochin Hospital, Paris, France) was already described [8]. All patients provided written informed consent, and the study was approved by the local ethics comitte (Scientific Comitte of Tumor Bank of Cochin Hospital) according to good clinical practice and applicable laws, and the declaration of Helsinki.

#### Animals

Mice were maintained in a 12-h light/dark cycle with ad libitum water and regular diet (65% carbohydrate, 11% fat, and 24% protein). All animal procedures reported herein were approved by the Paris-Descartes Ethical Committee for Animal Experimentation under protocol no. CEEA34.CP.077.12.

## Material and Reagents

Anti-6HisTag antibody was purchased from Life Technologies (Carlsbad, CA). Anti-β-actin was from Sigma-Aldrich (St-Louis, MO). Monoclonal mouse primary antibody against mouse glutamine synthetase was from BD Transduction Laboratories (le pont de Claix, France) and anti-Ki67 was from Leica Biosystems (Nanterre, France). The blood glucose monitor and associated test strips for glucose measurement were from Accu-Check Active, Roche Diagnostics (Basel, Switzerland). Recombinant human Wnt3a was obtained from R&D Systems (Minneapolis, MN).

### Plasmid Constructions

Sequences encoding a 3 glycine-linker and a 6 histidine-tag were added to that encoding the C-terminal part of *Homo sapiens* *Notum* using *Notum* cDNA (BC060882.1, kindly provided by Dr. M. Zebisch, University of Oxford, UK) as template and the following primers for PCR amplification: 5’-ATG GGC CGA GGG GTG CGC-3’ and 5’-**CTA***GTG GTG ATG GTG ATG ATG TCC GCC TCC*GCT TCC GTT GCT CAG CAT CCC CA-3’ as forward and reverse primers, respectively. The endogenous stop codon is replaced by a serine codon (underlined), the nucleotides in italics code for a 3 glycine-linker followed by a 6 Histidine-tag, and the new stop codon is indicated in bold. The resulting cDNA fragment, *Homo sapiens* Notum_3Gly_6His, was inserted into pDS_EF1-XB (kind gift from Dr. M. Lambert from Institut Cochin, Paris) by using Gateway^®^ technologies (Life Technologies) for mammalian expression to generate pET_HsNotum6His^WT^. The corresponding mutant, pET_HsNotum6His^S231A^, was obtained by site-directed mutagenesis with the QuikChange II Kit (Stratagene, La Jolla, CA, USA) and the primer 5’-TGC TGG CCG GGG CCA GCG CGG GGG-3’ and its reverse complement. All plasmid sequences were confirmed by DNA sequencing.

### Cell Culture and Transfections

The hepatocellular carcinoma cell line HuH7 was cultivated in Dubelcco’s modified Eagle’s medium (DMEM, Life Technologies) supplemented with 10% fetal bovine serum and 100U/ml penicillin/streptomycin at 37°C under 5% CO_2_. For transfection, 0.05x10^6^ cells were seeded in each well of a 24-well plate. Twenty four hours later, plasmids were added and the cells transfected by the Lipofectamine 2000 method according to the manufacturer’s protocol (Life Technologies). The knockdown of *NOTUM* was performed with a pool of four different siRNAs according to the manufacturer’s protocol (SMARTpool accell *NOTUM* siRNA Dharmacon, Lafayette, CO).

### Luciferase reporter assay

The TOPflash/FOPflash reporter plasmids (Millipore, Billerica, MA) were used to determine β-catenin-induced Tcf/Lef transcriptional activity. HuH7 cells were co-transfected in triplicate in accordance with the manufacturer’s instructions with the reporter plasmid and pRL-TK to normalize transfection efficiency. Forty-eight hours after transfection, Luciferase activity was measured with the Dual-Luciferase reporter assay system (Promega, Madison, WI).

### Mouse Models

The *Notum* targeting construct was generated from PCR products amplified from the DNA of 129/SV ES cells by Pfx polymerase (Life Technologies). A targeting vector was constructed: *LoxP* sites were introduced upstream from *Notum* exon2 and downstream from *Notum* exon8, and a hygromycin cassette flanked by *FRT* sites was introduced as a selection marker downstream from *Notum* exon8. The targeting construct was introduced by electroporation into embryonic stem cells from mouse strain 129/SV and selected on plates containing hygromycin. Appropriate clones were identified by PCR and confirmed by Southern blot analysis using 5’ and 3’ probes. Stem cells carrying the construct were injected into blastocysts from C57BL/6J mice to obtain chimeric mice. After germline transmission, mice carrying the targeted *Notum* allele were crossed with mice producing Flp recombinase to remove the hygromycin cassette resulting in mice carrying a *Floxed Notum* allele; these mice were then mated with C57BL/6J mice which express Cre recombinase under the control of the albumin gene promoter with α–fetoprotein enhancer (Alfp-Cre), which allows Cre recombinase expression at E9.5 specifically in hepatocytes [16]. For littermate genotyping, tail genomic DNA and primers P1 = (5’-TCT GAT CCT AGG CCA ACT CG-3’), P2 = (5’-GGT CCC AGA GCG TTG CCA GC-3’) and P3 = (5’-CCG GCT GTC CGG TGA GAT GC-3’) were used to verify the excision of DNA fragment which was in between the loxP sites. Generation of the tamoxifen-inducible model of acute β-catenin activation in the liver (APC^Δex14ex14^) is described in [4].

### Liver Samples and Histological Analysis

Mice were killed by cervical dislocation; some liver samples were snap-frozen in liquid nitrogen and stored at -80°C until analysis, and others fixed in neutral formalin buffer for 12h and then embedded in paraffin. Three-micron-thick sections were cut and stained in hematoxylin-eosin. RNA *in situ* hybridization was performed on cryostat sections (12 μm thick) as described in [4] with digoxygenin-labeled riboprobes and counterstaining in Nuclear Fast Red Solution (Sigma-Aldrich). The sections were counted with VectaMount and photographed with a color digital camera in a Zeiss photomicroscope. Immunostaining for glutamine synthetase (GS) and Ki67 antigen were performed as described in [17].

### Quantitative RT-PCR

Total RNA was isolated using TRIzol^®^ reagent according to the manufacturer’s instructions (Life Technologies). Reverse transcription was performed with 1 μg of total RNA using a Transcriptor First Strand cDNA Synthesis Kit (Roche Diagnostics) and random hexamer as primer. PCR amplification was performed using *Notum* Forward 5’-TTT ATG GCG CAA GTC AAG AG-3’ and *Notum* Reverse 5’-ACC AGT ACC TGT GCG TGT GT-3’ primers. Primers for PCR amplification of 18S, Axin2, Lect2, Arg, Glt1 are described in [4]. Real-time quantitative PCR was performed in triplicate on a LightCycler460 apparatus (Roche Diagnostics) and results are expressed relative to those for 18S rRNA.

### Whole Cell Extraction and Immunoblot Analysis

Cells were washed in ice-cold PBS, lysed in RIPA buffer (Sigma-Aldrich) containing 1X complete protease inhibitor cocktail (Roche Diagnostics) and centrifuged at 13,000 g for 10 min at 4°C. Supernatant (corresponding to the whole cell extract) was kept at -80°C for until analysis. Whole cell extracts were resolved by SDS-PAGE, transferred to nitrocellulose and blocked with 5% BSA. Blots were incubated with primary antibodies overnight at 4°C, washed, incubated with horseradish peroxidase-conjugated secondary antibodies for 1 h at room temperature, and washed further. The blots were developed by enhanced chemiluminescence (Pierce, Rockford, IL).

### Intra Peritoneal Glucose Tolerance Test

Mice were fasted for 16h and subjected to intraperitoneal glucose loading (2g glucose/kg). Blood glucose levels were measured at various times over the following 180 min.

### Insulin Dosage

Plasma insulin was assayed with a Mouse Ultrasensitive Insulin ELISA kit according to the manufacturer's guidelines (Alpco, Les Ulis, France)

### Statistical analysis

Student’s *t* test was used to determine the significance of differences between means. A *p* value of less than 0.05 was defined as significant and asterisks are used to denote significance as follows: *p<0.05, **p<0.01, ***p<0.001.

## Results

### *NOTUM* is overexpressed in human HCC with activating *CTNNB1* mutations and is a direct target of the Wnt/β-catenin pathway

**Fig 1:**
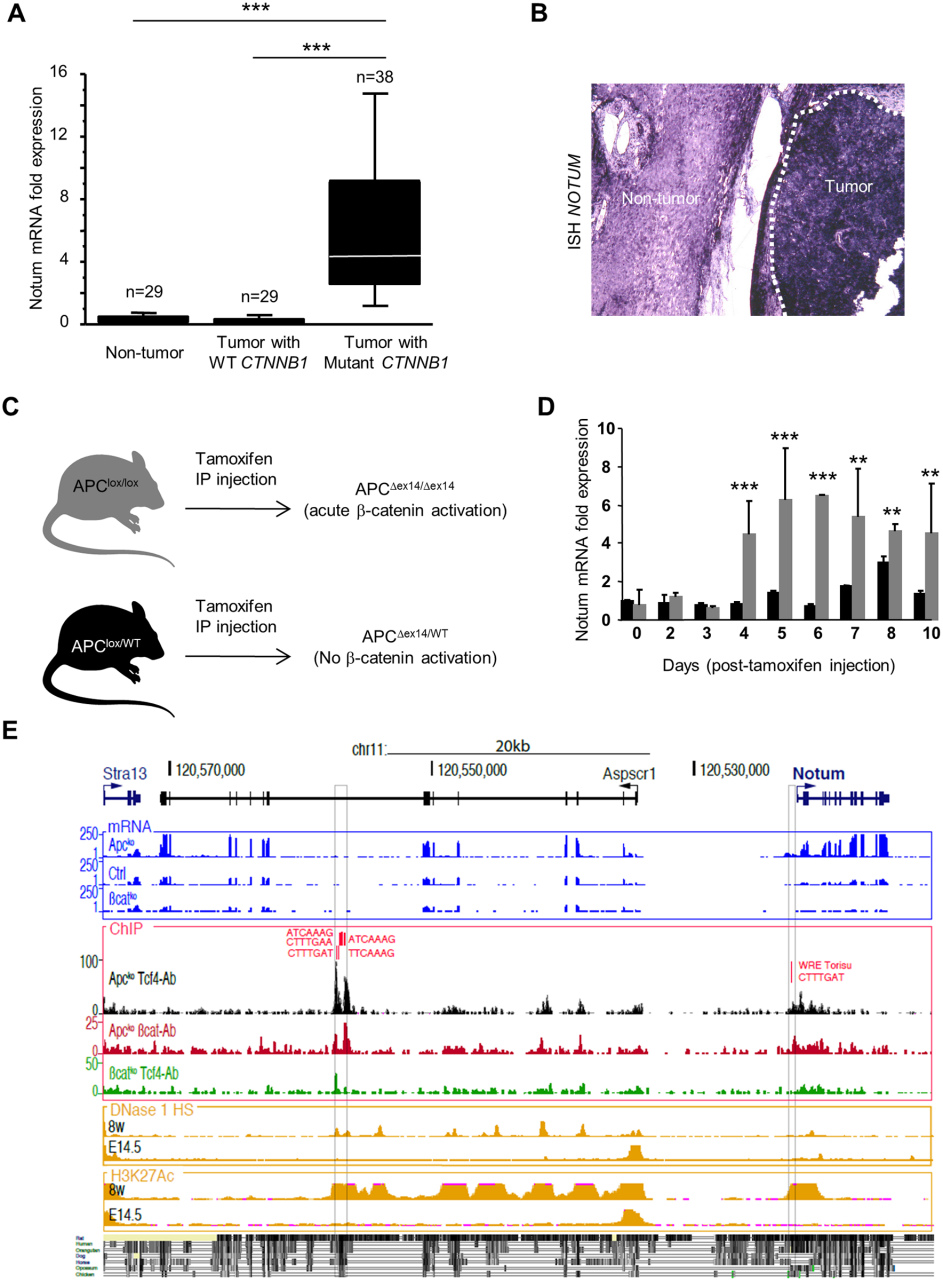
*Notum* is a positive target of β-catenin. (A) Total RNA was extracted from 96 samples (29 from HCC specimens with WT *CTNNB1*, 38 from HCC specimens with mutant *CTNNB1* and 29 from adjacent non tumor tissue that included 19 *CTNNB1*-mutated HCC and 10 non-mutated HCC). *Notum* mRNA was assayed by quantitative RT-PCR. (B) *NOTUM in situ* hybridization with mutantβ-catenin HCC specimens showed significantly stronger *NOTUM* labeling in tumor tissue than normal tissue. Seven HCC biopsies were analyzed and a representative result is shown. (C) Tamoxifen-inducible model of acute β-catenin activation in the liver. (D) Eight week-old mice were intraperitoneally injected with 0.3 mg tamoxifen and total RNA was extracted from WT liver (black rows) and from APC^Δex14/Δex14^ liver (grey rows) at the indicated times and *Notum* mRNA was assayed by quantitative RT-PCR. Results are normalized to those for 18S transcripts and the mean ± S.D. of triplicate samples is presented. (E) Genomic environment of the *Notum* gene (from mRNA-Seq and ChIP-Seq data described in [5]. Upper blue: mRNA-seq signals. ChIP-Seq signals are shown below with the presence within peaks of WREs depicted first in red; then in black, ChIP-Seq in *Apc*-ko conditions with an antibody against Tcf4; in red, ChIP-Seq in *Apc*-ko conditions with an antibody against β-catenin; in green, ChIP-Seq in β-catenin-ko conditions with an antibody against Tcf4. Below in yellow, ENCODE data for DNase 1 HS performed with 8-week-old and in E14.5 embryonic mouse livers (DNaseI Hypersensitivity by Digital DNaseI from ENCODE/University of Washington), of H3K27Ac marks in 8-week-old and in E14.5 embryonic livers (Histone Mods by ChIP-seq from ENCODE/LICR).

We performed microarray studies to identify genes specifically induced in HCC with activating mutations in *CTNNB1*. *NOTUM* was identified as one of the genes most up-regulated in response to β-catenin activation in HCC (data not shown). We assayed *NOTUM* mRNA by quantitative RT-PCR in a cohort of human HCC characterized for *CTNNB1* mutations [8]. *NOTUM* mRNA was 6.4 times more abundant in tumor than adjacent non-tumor tissue in HCC with mutant α-catenin; *NOTUM* mRNA did not differ in adjacent non-tumor tissue of HCC whatever of the *CTNNB1* mutation status of the tumor (Fig 1A). We performed *in situ* hybridization to analyze *NOTUM* expression at the cellular level: *NOTUM* was strongly overexpressed in tumoral hepatocytes (Fig 1B).

These findings suggest that β-catenin positively regulates *NOTUM* expression. To test this possibility, we studied *Notum* expression in the liver of mice with acute activation of the β-catenin signaling due to the inactivation of the negative regulator, the *Adenomatous polyposis coli* (*Apc*) gene in hepatocytes (Fig 1C)[4]. In this mouse model, *Notum* expression in liver of *Apc* mutant mice was five-fold higher than that in controls (Fig 1D). We recently identified two major binding sites for both β-catenin and TCF4, the nuclear partner of β-catenin, in our ChIP seq analysis that provided the repertoire of the nuclear binding response elements of β-catenin in hepatocytes [5]. One is in the promoter of the mouse *Notum* gene, in a region conserved between mouse and human [18]. The second β-catenin/TCF4 site is around 30 kbp upstream from the *Notum* transcription start site (Fig 1E). These observations indicate that *Notum* is likely a direct positive target gene of β-catenin in the mouse liver.

**Fig 2:**
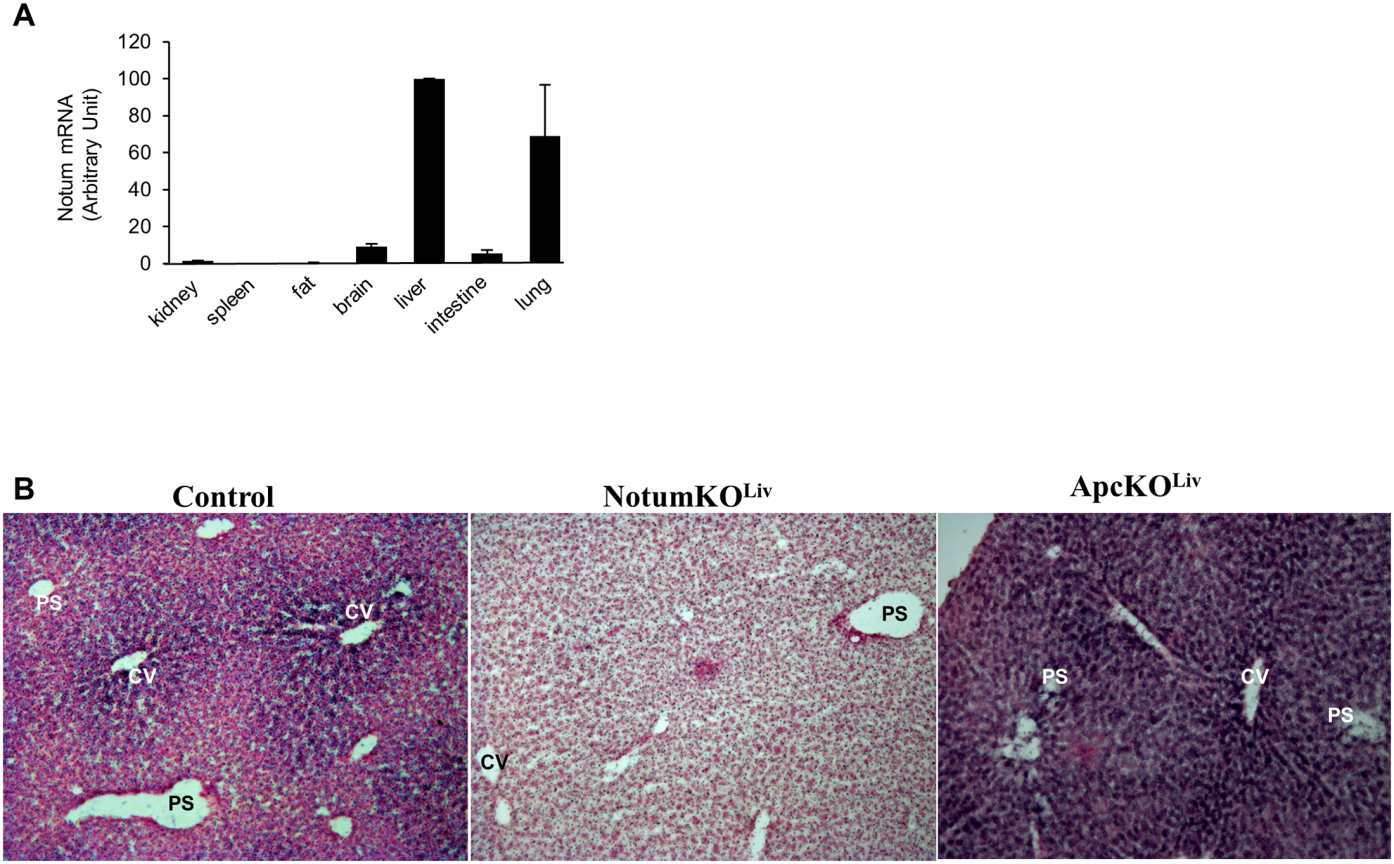
Pattern of *Notum* expression. (A) Total RNA was extracted from various mouse tissues and *Notum* mRNA assayed by quantitative RT-PCR. Results are normalized to those for 18S transcripts and the means ± S.D. of triplicate samples are presented. (B) Representative picture of *Notum in situ* hybridization with WT, NotumKO^Liv^ or APC^Δex14/Δex14^ mice liver. Portal spaces (PS) and hepatic centrilobular vein (CV) are indicated.

The expression pattern of *Notum* was then analyzed by quantitative RT-PCR in several adult mouse tissues (Fig 2A). *Notum* was mostly expressed in the liver and in the lung, with little expression in the brain, intestine or kidney. *In situ* hybridization showed that *Notum* expression was restricted to the perivenous region of the liver lobule, as expected for a liver β-catenin-positive target gene (Fig 2B).

### Control of β-catenin transactivation in HuH7 HCC cell line by *NOTUM*

**Fig 3:**
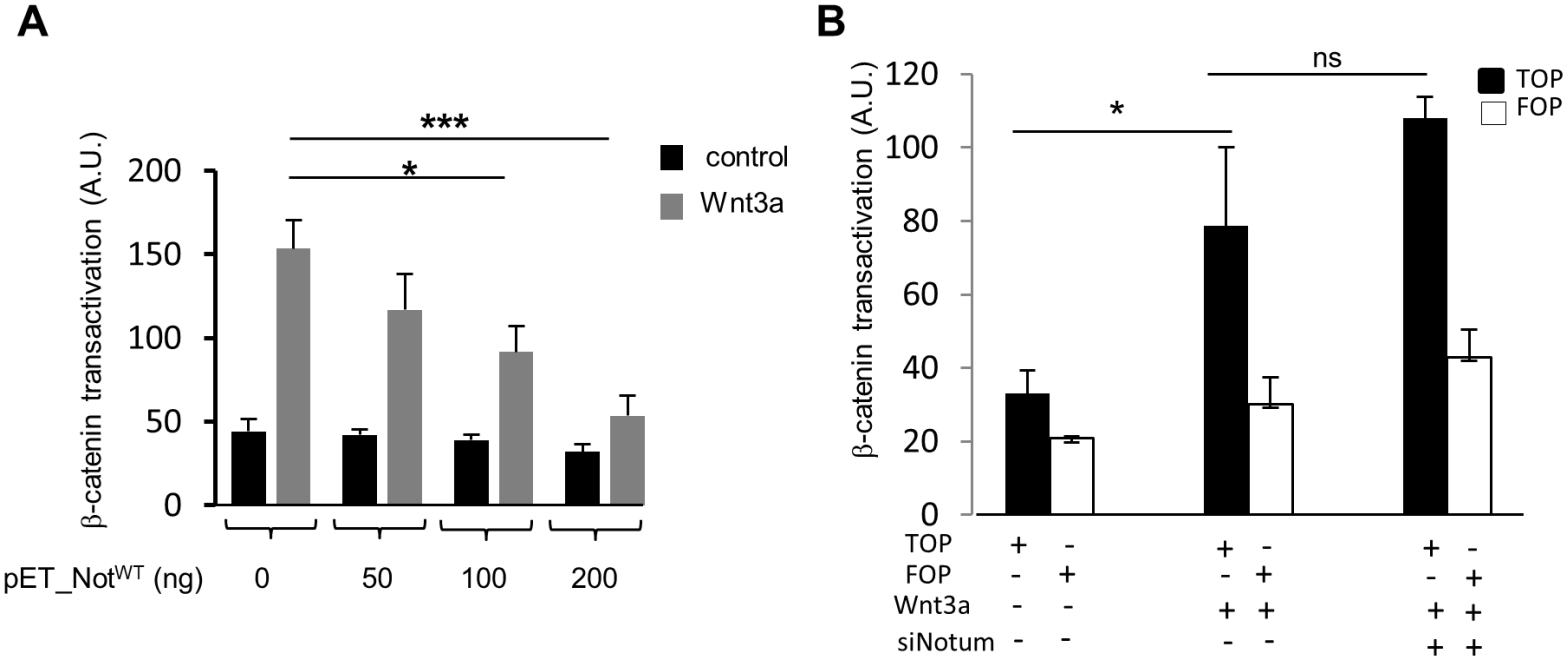
Regulation of Wnt3a/β-catenin signaling by *NOTUM* in HCC cell line Huh7. Huh7 cells were transfected with empty vector and pET-Notum6His as indicated, together with 50ng of TopFLASH *Firefly* Luciferase plasmid and 5ng of control *Renillia* Luciferase. Twenty-four hours after transfection, cells were stimulated or not with 50ng/ml recombinant human Wnt3a and analyzed for luciferase activity 24 hours later. Data represent means of three independent transfections with the standard deviations indicated. *NOTUM* silencing did not increase β-catenin activity. HuH7 cells were reverse-transfected as indicated with 100 pmol of *NOTUM* siRNA. Twenty four hours after, β-catenin luciferase reporters (TOPflash/FOPflash) were transfected and Wnt3a was added as indicated 16 hours post-transfection of the plasmids encoding luciferase. Luciferase activity was measured the following day.

The *Notum* gene is conserved across the animal kingdom, and is a negative regulator of Wnt/β-catenin signaling in several animal models [9, 10, 11,12]. To investigate the ability of mammalian *Notum* to modulate Wnt signaling in hepatocytes, we inserted human *NOTUM* into the mammalian expression vector pET. Transfected human HuH7 cells produced *Notum* that was able to inhibit a prototype Wnt responsive TOPFLASH reporter induced with recombinant Wnt3a protein, and this effect was dose dependent (Fig 3A). Therefore, like its counterparts in drosophila and in *C*. *elegans*, human *NOTUM* appears as a secreted protein which negatively regulates Wnt/β-catenin signaling. We then analyzed the effect of *NOTUM* knock-down by siRNA on β-catenin transactivation in HUH7 cells. Because *Notum* is a deacylase that removes palmiteoylation group from Wnt3a [14, 15], we tested the effect of *NOTUM* depletion in Wnt3a-treated cells. While β-catenin signaling was increased in response to Wnt3a stimulation, we did not observe any significant increase in β-catenin signaling after *NOTUM* knock-down by siRNA (Fig 3B). Altogether, our data demonstrate that *NOTUM* was able to control β-catenin activation when overexpressed, but the role of endogenous *NOTUM* in HuH7 remains unclear as its depletion by siRNA did not significantly increase β-catenin transactivation level.

### Generation of a conditional mouse model of *Notum* inactivation

**Fig 4:**
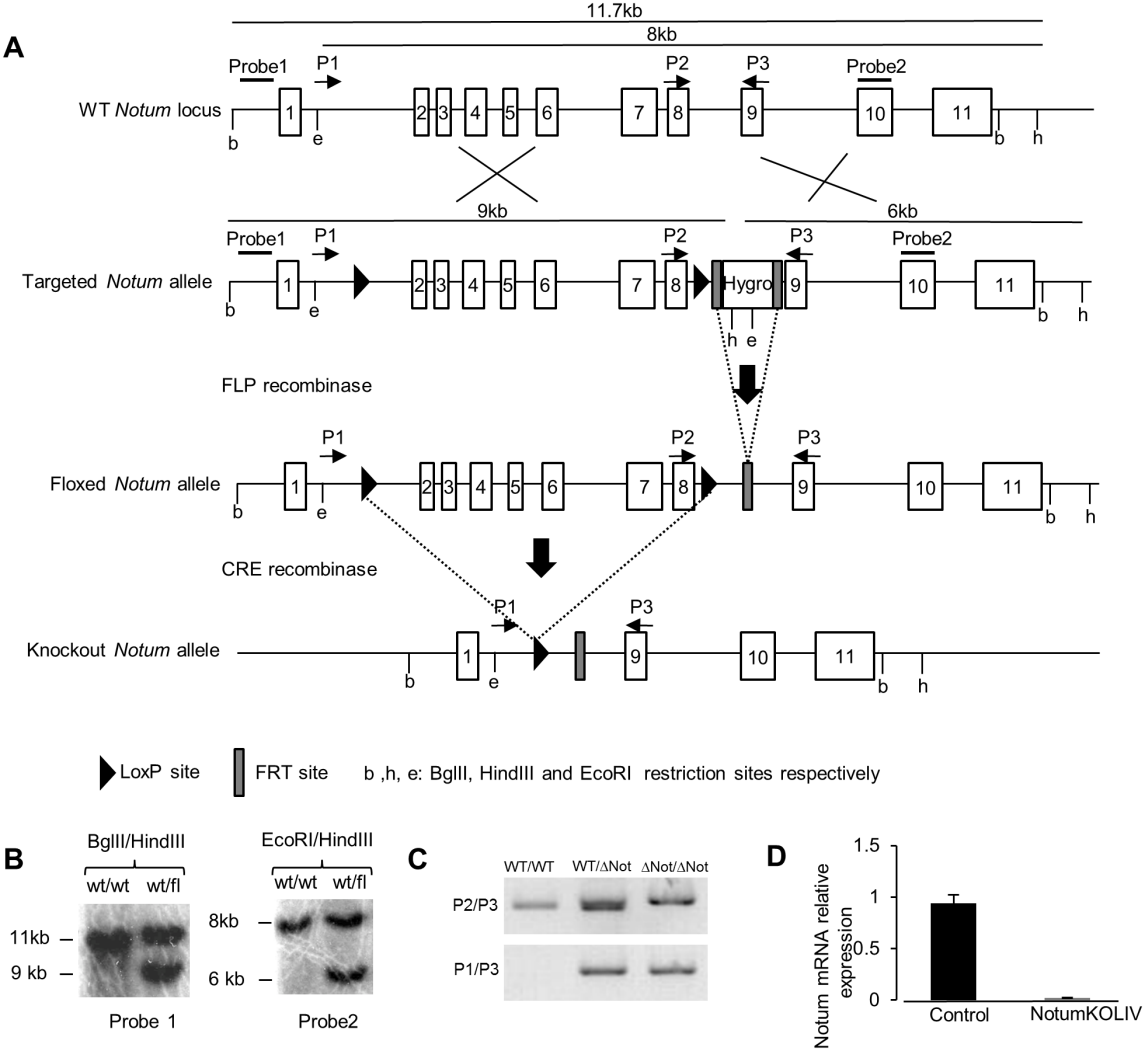
Generation and validation of conditional mouse model of *Notum* inactivation. (A) Diagram of the generation of the *Notum* conditional knockout. Structure of *Notum* locus with the targeted allele is shown. Numbered boxes indicate exons. Exons 2 to 8 were flanked by *loxP* sites (black arrowheads). A hygromycin-resistance cassette flanked by *FRT* sites was inserted downstream from the 3’ *loxP* site. The hygromycin-resistance cassette was excised by the expression of the FLP recombinase *in vivo*. Liver-specific disruption of exons 2 to 8 flanked by *loxP* sites was achieved by crossing *Notum*^*WT/lox*^ mice with Alfp-Cre mice. (B) Genomic DNA from *Notum*^*WT/WT*^ or *Notum*^*WT/lox*^ mice was digested with BglII/HindIII or EcoRI /HindIII and was analyzed by Southern blot using probe1 or probe2, respectively. (C) Genotyping by PCR of liver DNA from: control (*Notum*^*WT/WT*^), *Notum*^WT/ΔNot^, and *Notum*^*KOLiv*^ *(Notum*^ΔNot/ΔNot^) mice, with the P1, P2 and P3 primers shown in A. (D) Total RNA was extracted from liver of *Notum*^*WT/WT*^ (control) and *Notum*^*KOLiv*^ E16.5 embryos and assayed by quantitative RT-PCR for *Notum* mRNA.

We investigated the possible role of *Notum* in the liver patterning involved in metabolic zonation. We produced a conditional mouse model of *Notum* inactivation to test whether deletion of *Notum* in the hepatocytes of adult mice affected metabolic zonation (Fig 4A). We produced mice carrying *Notum* alleles in which the sequence from exon 2 to exon 8 is flanked by *LoxP* sites. Southern blotting with positive embryonic stem cell clones confirmed that the homologous recombination of the targeted allele was as intended (Fig 4B). These clones were injected into blastocyts to obtain chimeric mice. After germline transmission, *Notum*^lox/lox^ mice were produced using C57BL/6J mice expressing FLP recombinase to remove the hygromycin selection cassette and mated with C57BL/6J until backcross N3. *Notum*^lox/lox^ mice were then mated with Alfp-Cre mice, allowing Cre recombinase expression in hepatoblasts, from embryonic stage E10.5 [16]. PCR of liver genomic DNA with various primers confirmed that the sequence between *Notum* exon 2 and exon 8 had been deleted in the liver of *Notum*^lox/lox^ Alfp-Cre mice; these mice were named *Notum*KO^Liv^ (Fig 4C). Invalidation of *Notum* expression was confirmed at the mRNA level by RT-qPCR with samples from E16.5 embryo livers of *Notum*KO^Liv^ (Fig 4D) and by *in situ* hybridization. No *Notum* mRNA was found in liver sections from adult *Notum*KO^Liv^ mice whereas *Notum* mRNA was detected across the whole lobule in the liver of mice with constitutive β-catenin signaling in hepatocytes (*Apc* mutant mice) (Fig 2B).

These various findings confirm the deletion of *Notum* from the liver of *Notum*KO^Liv^ mice.

### Deletion of *Notum* in the liver did not affect liver architecture or liver zonation

**Fig 5:**
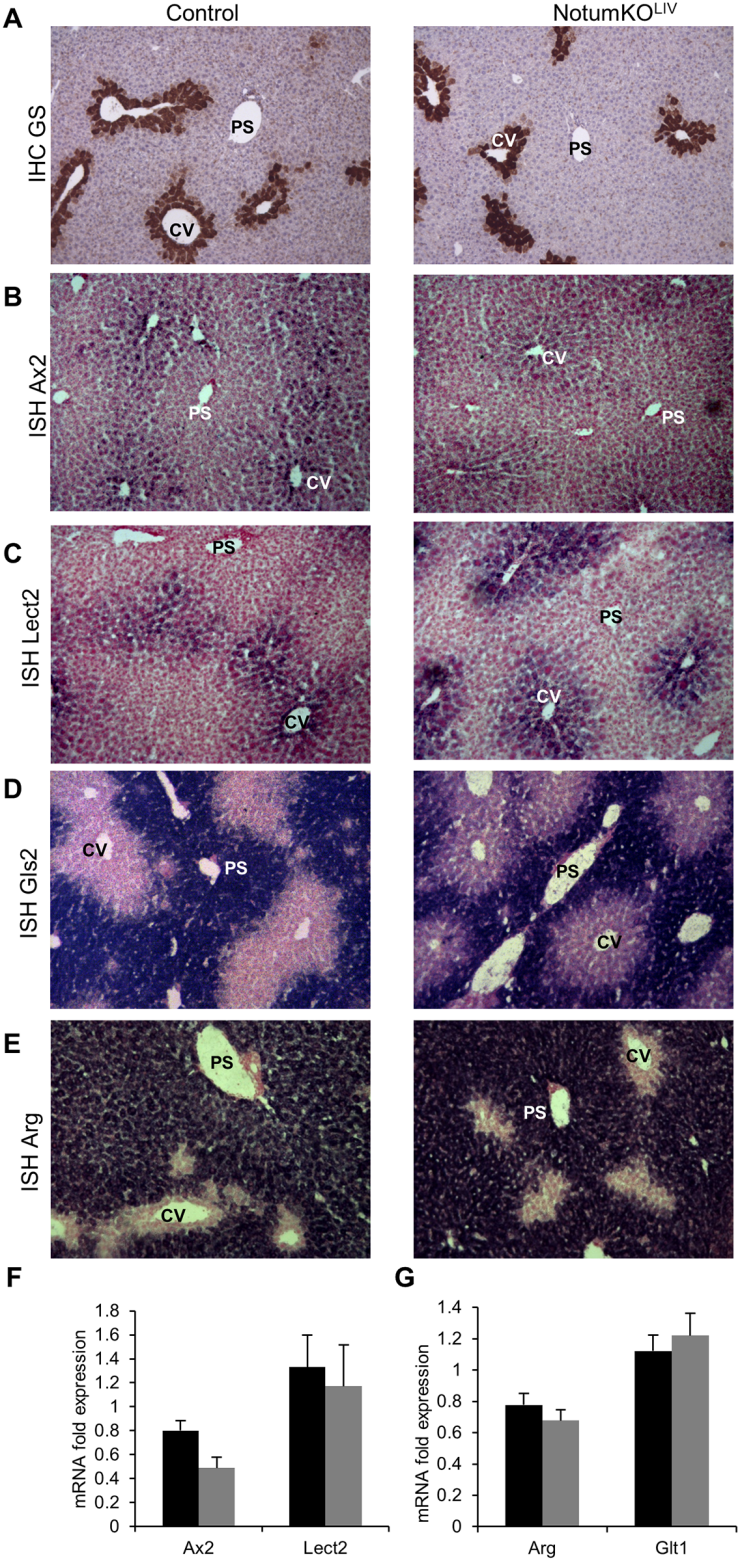
*Notum* inactivation did not modify liver metabolic zonation. (A-B-C-D-E) Glutamine synthetase immunostaining, and Axin2 and Lect2 Gls2 and Arg *in situ* hybridization were performed on *Notum*^*WT/WT*^ and *Notum*^*KOLiv*^ liver samples. Representative pictures are shown. Portal spaces (PS) and hepatic centrilobular vein (CV) are indicated. (F-G) Quantitative RT-PCR analysis of Axin2, Lect2, Arg and Glt1 mRNAs in *Notum*^*WT/WT*^ (n = 4, black rows) and liver *Notum*^*KOLiv*^ (n = 5, grey rows); values were normalized to those for 18S transcripts level, and means ± S.E.M are reported.

*Notum*KO^Liv^ mice were born in Mendelian proportion and did not display any macroscopic phenotype until adult. Liver weight and liver to body ratio were similar to those for wild-type mice. H&E staining and proliferation studies of liver sections did not show any major defect in *Notum*KO^Liv^ mice (data not shown). We analyzed liver zonation in the hepatocyte-specific *Notum* knockout mice by *in situ* hybridization: in the mutant mice, expression of the β-catenin positive targets Glutamine synthetase, Axin2 and Lect2 were restricted to pericentrilobular hepatocytes whereas expression of the negative targets Gls2 and Arg were restricted to periportal hepatocytes; this pattern is not different from that in the controls (WT liver) indicating no modification of liver zonation (Fig 5A and 5E). This was confirmed by analysis of the abundance of the mRNAs by RT-qPCR, which provided no evidence of difference between the mutant and the control animals (Fig 5F and 5G).

### Deletion of *Notum* in the liver significantly increases the risk of obesity with age

**Fig 6:**
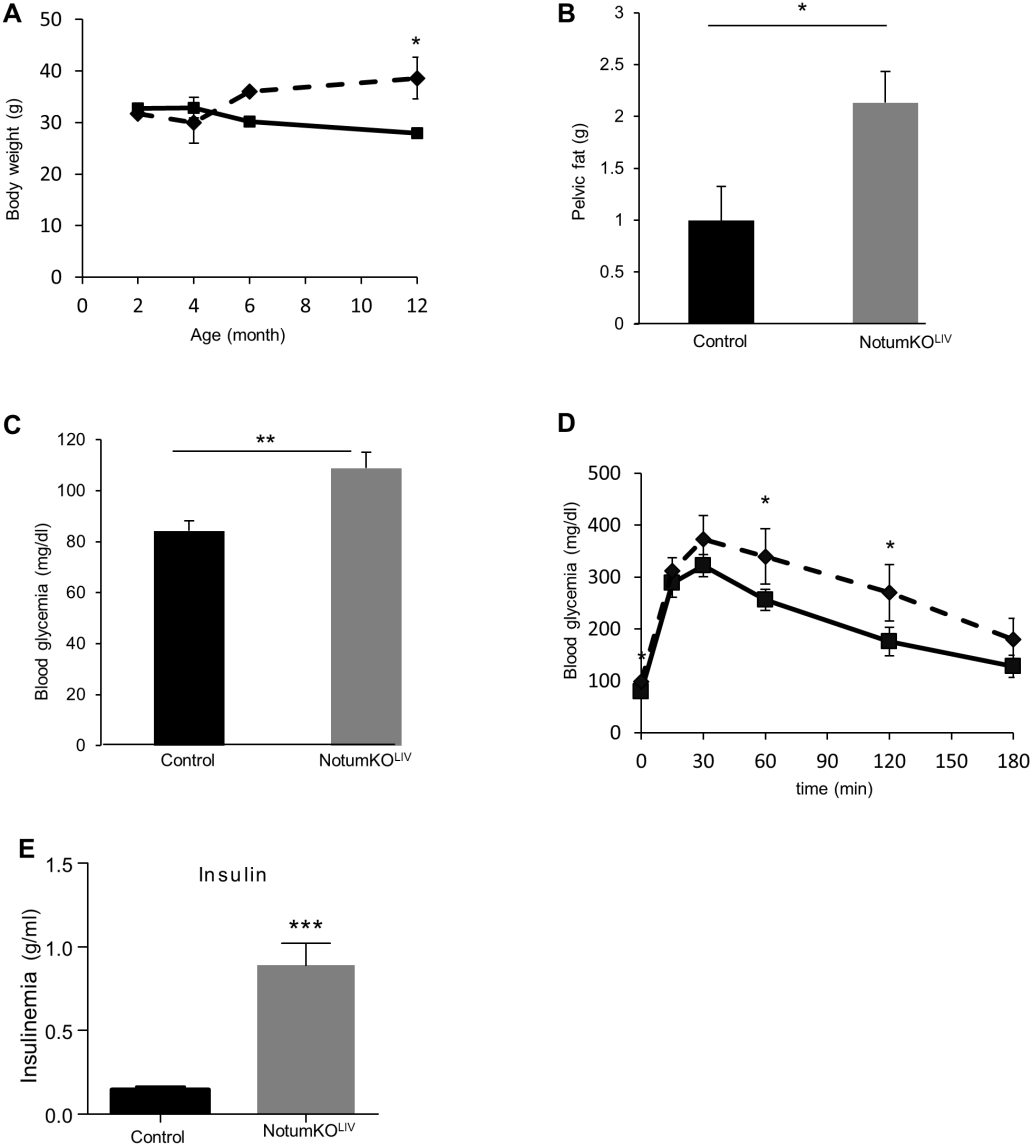
*Notum* depletion in the liver increases risk of age-related obesity in males. (A) Male *Notum*^*WT/WT*^ (n = 8, plain line) and *Notum*^*KOLiv*^ (n = 5, dashed line) mice were weighed at various adult ages. Body weight is presented as means ± S.E.M. (B) Pelvic fat pads were harvested from 1 year-old *Notum*^*WT/WT*^ (n = 6) and *Notum*^*KOLiv*^(n = 6) mice and weighed. (C) Blood glycemia was measured in 1 year-old *Notum*^*WT/WT*^ and mice. (D) Intraperitoneal glucose tolerance tests were performed on 1 year-old *Notum*^*WT/WT*^ (n = 6, plain line) and *Notum*^*KOLiv*^ (n = 6, dashed line) mice. Experiments were performed in duplicate and means ±S.E.M. are shown. (E) Plasma insulin concentrations in fasted mice.

The published evidence suggests that *Notum* may be involved in fine tuning regulation of Wnt/β-catenin signaling rather than “on/off” regulation. Consequently, we tested for the effects of *Notum* depletion in the liver later during the lives of the mice. We analyzed the phenotype of *Notum*KO^Liv^ mice at 4, 6, 9 and 12 months of age. We have noticed no difference in the liver histology between old WT and KO mice. In addition, the metabolic zonation and Wnt/β-catenin signaling in *Notum*KO^Liv^ mice at these different ages were indistinguishable from those in the wild-type (data not shown). However, numerous *Notum*KO^Liv^ males displayed significant weight gain from 6 months of age, such that the mean weight of the males was 38.6 g at 12 months (versus 27.9 g for their WT littermates, Fig 6A). The liver to body ratio was unaffected (3.48±0.12% for the WT versus 3.70±0.15% for *Notum*KO^Liv^), but there was substantial excess of pelvic fat in 12 month-old *Notum*KO^Liv^ mice (Fig 6B), and the fasting blood glucose concentration was significantly elevated (108.9±6.1 mg/dl for one year old *Notum*KO^Liv^ vs 84.2±4.1 mg/dl in WT littermates; Fig 6C). *Notum*KO^Liv^ mice were glucose intolerant, and had very high plasma insulin concentrations after 16h of fasting (0.89 ± 0.12 ng/ml versus 0.15 ± 0.02 ng/ml for their WT littermates) (Fig 6D and 6E). Intriguingly, *Notum*KO^Liv^ females displayed unchanged body weight, pelvic fat and fasting blood glucose concentration when compared to WT female littermates (data not shown). Thus, our results indicate a sexual dimorphism in *Notum*KO^Liv^ mice with metabolic disorders that appeared specifically in males with age.

## Discussion

In this study, we show that *NOTUM* expression is strongly up-regulated in human HCC, specifically in the subclass of HCC carrying an activating mutation of *CTNNB1*. This is consistent with the report by Torisu et al. [18]. We used the conditional *Apc*^Δ14^ mouse model of acute activation of β-catenin signaling in the liver to confirm that *Notum* was positively regulated by β-catenin in that organ. *In situ* hybridization of WT liver sections showed that *Notum* expression is restricted to pericentrilobular hepatocytes, which is the zone where β-catenin signaling is active in the physiological liver [4]. However, *Notum* expression extended to the whole lobule in *Apc*-deficient liver. ChIP seq experiments identified two TCF4-binding sites in the *Notum* regulatory region. Our findings are thus consistent with *Notum* being a direct target of β-catenin in mouse liver.

We confirmed that *NOTUM* can inhibit Wnt/β-catenin signaling *in vitro* with the overexpression of recombinant *NOTUM* in a Wnt3a-stimulated hepatocyte cell line, HuH7. But *in vitro* knock-down of *Notum* in Wnt-3a-treated HuH7 cells failed to increase β-catenin activity showing that the control of the β-catenin signaling is more complex than anticipated. Accordingly, analyses with the *Notum* conditional mouse model we generated did not reveal any modification of liver zonation *in vivo* in the mutant animals. Even though *Notum* expression was completely abolished in hepatocytes, at least from E16.5, liver development was unaffected in mutant mice and they did not display any phenotype until adults. We cannot rule out the possibility that *Notum* is involved at earlier stages of liver development, but *Notum* does not appear to be required during the late stage of liver development. In addition, liver metabolic zonation remained unchanged in mutant mice, at least until one year of age. Altogether, our results argue either that in the liver, *NOTUM* do not control Wnt/β-catenin signaling or that complex redundant mechanisms control and maintain Wnt activity.

Finally, we observed a progressive increase in body weight with age associated with a significant increase in blood glycemia, glucose intolerance and insulinemia in male *Notum* mutant mice, relative to control littermates. This suggests that male *Notum*KO^Liv^ mice are at high risk of developing metabolic disorders such as diabetes. Although liver from *Notum*KO^Liv^ did not display any phenotype, the hepatic loss of secreted *NOTUM* may affect peripheral organs that are also contributors to insulin resistance. Notably, a substantial excess of pelvic fat was observed in *Notum*KO^Liv^. Thus, peripheral tissue (at least adipose tissue) may contribute to the pathophysiological mechanisms resulting from the hepatic loss of *Notum*.

Several genome wide association studies indicate that several components of the Wnt signaling pathway including TCF7L2, the nuclear partner of β-catenin, are genetic determinant of type 2 diabetes (T2D) risk in humans[19] [20]. However, our results questioned the fact that *NOTUM* is a component of the Wnt/β-catenin pathway in the liver. Regardless of the potential link of *NOTUM* to the Wnt pathway, *NOTUM* appears as a new susceptibility factor to diabetes and obesity. It reveals also that it is important to look outside the pancreas to understand the mechanisms linking genetic variation and T2D risk [21] [22].

Importantly, we observed that the obesity and glucose intolerance developed in *Notum*KO^Liv^ mice are gender-specific, the metabolic phenotype was not present in *Notum*KO^Liv^ old female. Sexual dimorphism in obesity is well described in humans [23]. While the connection between obesity and diabetes is well established in men, it is less so for women and the mechanism underlying these sexually dimorphic influences remain poorly understood. Although not investigated, *NOTUM* might be a new factor responsible for sex differences in metabolic disorders.

In conclusion, we report that *Notum* is strongly expressed by hepatocytes and is a direct target of the Wnt/β-catenin signaling in liver. Disruption of its expression in the liver does not have significant consequences on liver development and metabolic zonation. We also show that obesity and deregulation of glucose homeostasis appeared progressively with age specifically in male *Notum*-deficient mice, implicating *NOTUM* in the physiopathology of obesity and T2D in a gender-specific manner.
